# Complete Genome Sequences of 13 Human Respiratory Syncytial Virus Subgroup A Strains of Genotypes NA1 and ON1 Isolated in the Philippines

**DOI:** 10.1128/genomeA.00151-18

**Published:** 2018-03-08

**Authors:** Rungnapa Malasao, Yuki Furuse, Michiko Okamoto, Clyde Dapat, Mayuko Saito, Mariko Saito-Obata, Raita Tamaki, Edelwisa Segubre-Mercado, Socorro Lupisan, Hitoshi Oshitani

**Affiliations:** a Department of Virology, Tohoku University Graduate School of Medicine, Sendai, Japan; b Department of Microbiology, Faculty of Medicine, Chiang Mai University, Chang Mai, Thailand; c Frontier Research Institute for Interdisciplinary Sciences, Tohoku University, Sendai, Japan; d Institute for Frontier Life and Medical Sciences, Kyoto University, Kyoto, Japan; e Hakubi Center for Advanced Research, Kyoto University, Kyoto, Japan; f RITM-Tohoku Collaborating Research Center on Emerging and Reemerging Infectious Diseases, Muntinlupa, Philippines; g Research Institute for Tropical Medicine, Muntinlupa, Philippines

## Abstract

Complete genome sequences of 13 human respiratory syncytial virus strains were determined from samples obtained from children hospitalized in the Philippines between 2012 and 2013 because of acute respiratory infection. We identified amino acid polymorphisms between the NA1 and ON1 genotypes in the P, G, F, and L proteins.

## GENOME ANNOUNCEMENT

Human respiratory syncytial virus (HRSV) is a major cause of acute lower respiratory tract infection in infants and young children (1). Currently, no vaccine against HRSV is commercially available; the only pharmaceutical option is a monoclonal antibody used for prophylactic treatment of high-risk children (2). Among the HRSV subgroup A viruses, at least 11 genotypes have been identified, including GA1–7, SAA1, NA1, NA2, and ON1 (3, 4). The ON1 genotype, which has a 72-nucleotide duplication in the G gene, was first identified in 2012, and it has replaced its ancestor genotype, NA1, as the predominant genotype in several parts of the world, including the Philippines (5, 6).

We obtained nasopharyngeal swab samples from children hospitalized in the Philippines between 2012 and 2013 because of acute respiratory infection and isolated HRSV strains after culturing the swab samples in HEp-2 cells (5). Thirteen isolates were selected for whole-genome sequencing in the present study. Viral RNA was extracted from culture supernatants using the QIAamp MinElute virus spin kit (Qiagen, Hilden, Germany), and cDNA was transcribed using the SuperScript III reverse transcriptase and random primers (Thermo Fisher Scientific, Waltham, MA). Whole-genome sequences were obtained from 18 overlapping PCR fragments, produced using HRSV-specific primers (7–9). The PCR products were purified using the illustra ExoProStar digestion kit (GE Healthcare, Little Chalfont, UK). Sanger sequencing was subsequently performed using the BigDye Terminator version 3.1 cycle sequencing kit and the 3730 DNA analyzer instrument (Thermo Fisher Scientific, Waltham, MA). Informed consent was obtained from the guardians of all participants. The study protocol was approved by the Ethics Committee of the Tohoku University Graduate School of Medicine, Japan, and the Institutional Review Board of the Research Institute for Tropical Medicine, Philippines.

Among the 13 sequenced isolates, 6 were identified as genotype NA1 and 7 as genotype ON1, both of which belong to HRSV subgroup A. The complete genome sequences of the NA1 and ON1 strains were 15,204 and 15,277 nucleotides long, respectively. As previously reported, the ON1 strains had a 72-nucleotide duplication in the second hypervariable region of the G gene (3). Moreover, we identified a single nucleotide insertion in the ON1 genome located in the untranslated region between the SH and G genes. Comparison of the NA1 and ON1 genomes revealed nonsynonymous substitutions in the genes encoding the P protein (position 92), G protein (positions 126, 130, 232, 237, 253, and 314), F protein (position 104), and L protein (positions 598 and 835).

The reason the ON1 strain has replaced the previously circulating NA1 strain and has become a globally predominant genotype remains unclear (6). The complete genome sequences of the recently circulating strains reported in this study may provide some insights for understanding the genetic diversity and evolution of HRSV.

### Accession number(s).

The complete genome sequences of the 13 HRSV strains in this study have been deposited at GenBank under the accession numbers KY654506 to KY654518.
